# Study of Factors Affecting Ureteral Access Sheath Insertion in Retrograde Intrarenal Surgery

**DOI:** 10.7759/cureus.112430

**Published:** 2026-07-10

**Authors:** Siddanagouda B Patil, Santosh Patil, Vinay S Kundargi, Anupam Banerjee, Karthik M Chavannavar

**Affiliations:** 1 Urology, Shri B.M. Patil Medical College, Hospital and Research Centre, Bharatiya Lingayat Development Educational (BLDE) (Deemed to be University), Vijayapura, IND

**Keywords:** body mass index, retrograde intrarenal surgery, ureteral access sheath, ureteric orifice morphology, urolithiasis

## Abstract

Introduction: Urolithiasis is a common urological disorder with a high recurrence rate and increasing global burden. Retrograde intrarenal surgery (RIRS) is an established minimally invasive treatment for renal and upper ureteric calculi. The ureteral access sheath (UAS) facilitates repeated access, improves irrigation, reduces intrarenal pressure, and enhances procedural efficiency. However, successful UAS insertion remains technically challenging and may influence surgical outcomes. The present study aimed to evaluate demographic, anthropometric, anatomical, and stone-related predictors of successful UAS insertion during RIRS.

Materials and methods: This prospective observational study included 120 patients undergoing RIRS for renal or upper ureteric calculi. Demographic, clinical, radiological, and intraoperative variables were analyzed, and patients were categorized into successful and failed UAS insertion groups. Variables assessed included age, gender, body mass index (BMI), stone characteristics, hydronephrosis, ureteric orifice morphology, and computed tomography (CT)-measured common iliac artery diameter. Statistical analysis was performed using the Chi-squared test and the independent t-test.

Results: Successful UAS insertion was achieved in 104 (86.7%) patients, while failure occurred in 16 (13.3%). Age and gender were not significantly associated with UAS insertion outcome (p > 0.05). Patients with failed insertion had significantly higher body weight and BMI (p < 0.001). Obesity (BMI ≥ 30 kg/m²) was strongly associated with insertion failure (χ² = 47.96, p < 0.0001). All failed insertions occurred in patients with round-shaped ureteric orifices, whereas tent-shaped ureteric orifices were observed exclusively in the successful insertion group (p < 0.001). The mean CT-measured common iliac artery diameter was significantly greater in failed cases (p < 0.001). Stone characteristics and hydronephrosis were not significant predictors.

Conclusion: Successful UAS insertion during RIRS is predominantly influenced by anthropometric and anatomical factors rather than stone-related variables. Higher BMI, round-shaped ureteric orifice morphology, and increased CT-measured common iliac artery diameter are important predictors of failed UAS insertion and may aid in preoperative risk assessment and surgical planning.

## Introduction

Urolithiasis is a major global health problem with an estimated prevalence ranging from 1% to 15% worldwide, and its incidence has increased steadily over the past few decades due to changing dietary habits, sedentary lifestyle, obesity, and metabolic disorders [1,2]. The disease is characterized by a high recurrence rate, with approximately one-third to one-half of affected individuals experiencing recurrent stone formation within five years [2,3]. In developing countries such as India, the burden of urinary stone disease is further intensified by delayed diagnosis, limited accessibility to advanced endourological care, and regional environmental and dietary influences [4]. Renal calculi account for a substantial proportion of stone disease requiring intervention and frequently necessitate minimally invasive surgical management for effective stone clearance [5].

Retrograde intrarenal surgery (RIRS) has evolved into one of the most preferred minimally invasive procedures for the management of renal and upper ureteric calculi, particularly stones measuring ≤20 mm [6]. Advancements in flexible ureteroscopes, laser lithotripsy systems, and accessory instruments have significantly improved procedural efficacy and safety, leading to wider acceptance of RIRS in contemporary urological practice [7]. The ureteral access sheath (UAS) has become an integral component of RIRS as it facilitates repeated entry into the collecting system, improves irrigation drainage, reduces intrarenal pressure, enhances visualization, and may reduce operative time and infectious complications [8]. Consequently, UAS placement is routinely attempted in most RIRS procedures, especially in patients with larger stone burden or anticipated prolonged operative duration [9].

Despite its advantages, successful insertion of the UAS remains technically challenging and may not always be feasible, particularly during primary RIRS without prior ureteral stenting [10]. Failure of UAS placement can result in abandonment of the procedure, requirement of pre-stenting with delayed surgery, conversion to alternative procedures, or performance of sheathless ureteroscopy, thereby increasing treatment cost, patient morbidity, and operative complexity [11]. In addition, forceful advancement of the sheath may lead to ureteral wall injury, mucosal ischemia, hematuria, postoperative pain, and long-term ureteral stricture formation [12]. Several demographic, anthropometric, stone-related, and anatomical variables have been proposed to influence the ease of UAS insertion, including age, sex, body mass index (BMI), ureteral compliance, stone characteristics, hydronephrosis, ureteric orifice morphology, and computed tomography (CT)-based anatomical parameters such as common iliac artery diameter [8,13].

Although previous studies have evaluated the outcomes and safety of UAS use during RIRS, literature specifically addressing preoperative predictors of successful UAS insertion remains limited and inconsistent, particularly in the Indian population [14,15]. Identification of such predictors may facilitate appropriate patient selection, preoperative counselling, operative planning, consideration of pre-stenting in high-risk patients, and reduction of ureteral injury. Therefore, the present study aimed to evaluate the demographic, anthropometric, anatomical, and stone-related factors, including CT-measured common iliac artery diameter, affecting the successful insertion of the UAS during RIRS.

## Materials and methods

This prospective observational study was conducted in the Department of Urology at Bharatiya Lingayat Development Educational (BLDE) (Deemed to be University), Shri B. M. Patil Medical College, Hospital and Research Centre, Vijayapura, Karnataka, India, over a study period extending from July 2024 to March 2026. The study was undertaken after obtaining approval from the Institutional Ethics Committee (IEC No.: BLDE (DU)/IEC-SBMPMC/150/2023-24), and written informed consent was obtained from all participants prior to enrollment. Adult patients undergoing RIRS for renal or upper ureteric calculi constituted the study population.

The sample size for the present study was calculated based on the prevalence of failed UAS insertion reported by Hu et al., in which 11.2% of patients experienced failure of UAS insertion during RIRS [11]. The sample size was estimated using the formula:

$n = \frac{Z^{2} \times p \times q}{d^{2}}$

where *n* represents the required sample size, *Z* is the standard normal variate corresponding to the 95% confidence interval (1.96), *p* is the estimated prevalence of failed UAS insertion (11.2%), *q* = 1 − *p*, and *d* is the allowable error (6%). Substituting these values, the minimum required sample size was calculated to be 106 patients. Considering possible dropouts and incomplete data, a final sample size of 120 patients was included in the study.

Patients aged >18 years with renal or upper ureteric calculi measuring up to 2 cm on CT were included in the study. Patients with acute pyelonephritis, congenital or acquired urinary tract anatomical abnormalities precluding standard RIRS, or a history of previous urological intervention were excluded. Patients with previous urological intervention were excluded to eliminate the potential influence of prior ureteral dilatation or ureteral stenting on the success of UAS insertion.

All enrolled patients underwent detailed preoperative evaluation using a structured proforma. Baseline demographic data, including age, sex, height, body weight, and BMI, clinical history, and radiological findings were documented. Routine preoperative investigations included complete blood count, blood glucose, serum creatinine, serum electrolytes, urine routine examination, urine culture and sensitivity, electrocardiography, chest radiography, and CT of the kidney-ureter-bladder region, including plain and contrast-enhanced studies. Stone-related parameters including stone size, stone density, stone location, hydronephrosis, ureteric orifice morphology, and CT-measured ipsilateral common iliac artery diameter were assessed preoperatively.

Ureteric orifice morphology was assessed intraoperatively during cystoscopy by a single experienced consultant urologist and categorized as either round-shaped or tent-shaped according to its visual appearance. All procedures were performed by the same surgeon using a standardized operative protocol, thereby minimizing interobserver variability in the assessment of ureteric orifice morphology and surgical outcomes.

All procedures were performed under general or regional anesthesia (spinal or epidural) with the patient in the lithotomy position. A 0.035-inch hydrophilic guidewire was advanced into the renal pelvis under fluoroscopic guidance, following which a semi-rigid or flexible ureteroscope was introduced to inspect the ureter. A commercially available UAS of appropriate size (10/12 Fr and 9.5/11.5 Fr, as clinically indicated) was used according to the institutional protocol under fluoroscopic guidance. In cases where initial ureteroscopic passage was unsuccessful, sequential fascial dilators were used to facilitate ureteral dilatation before attempting UAS insertion. Initial insertion was attempted using a 10/12 Fr UAS; when advancement was unsuccessful despite adequate dilatation, a smaller 9.5/11.5 Fr sheath was subsequently attempted. If adequate ureteral dilatation could not be achieved despite sequential dilatation, the procedure was abandoned and managed with double-J stenting or conversion to percutaneous nephrolithotomy. Patients requiring pre-stenting or conversion to an alternative procedure after unsuccessful UAS insertion were classified as having failed UAS insertion.

Patients were subsequently stratified into two groups based on intraoperative findings. Group 1 comprised patients with successful UAS insertion, with or without prior fascial dilatation, whereas Group 2 comprised patients in whom UAS insertion failed.

The primary outcome measure was successful insertion of the UAS. Secondary outcome measures included the association of demographic variables, anthropometric parameters, stone characteristics, hydronephrosis, ureteric orifice morphology, and CT-based anatomical parameters, including ipsilateral common iliac artery diameter, with UAS insertion outcome.

All collected data were entered into Microsoft Excel (Microsoft Corp., Redmond, WA, USA) and analyzed using IBM SPSS Statistics version 26 (IBM Corp., Armonk, NY, USA). Continuous variables were expressed as mean ± standard deviation, whereas categorical variables were presented as frequencies and percentages. Continuous variables were compared using the independent t-test, and categorical variables using the Chi-squared test. Comparisons involving more than two groups were performed using one-way analysis of variance (ANOVA). Effect sizes were reported as Cohen's d for independent t-tests, Cramer's V for Chi-squared tests, and eta-squared (η²) for ANOVA. A p-value of <0.05 was considered statistically significant.

## Results

Out of 120 patients included in the study, successful insertion of the UAS was achieved in 104 (86.7%) patients, while failure to insert the sheath was observed in 16 (13.3%) patients, indicating an overall UAS insertion success rate of 86.7% during RIRS (Table 1).

**Table 1:** Outcome of Ureteral Access Sheath (UAS) Insertion (n = 120) Data are presented as number (percentage). Statistical significance: p-value < 0.05 was considered statistically significant.

Outcome	n (%)
Successful insertion	104 (86.7%)
Failure to insert	16 (13.3%)
Total	120 (100.0%)

The majority of patients belonged to the 31-40 years age group (42 (35.0%)), followed by the 51-60 years age group (30 (25.0%)). Successful UAS insertion was most commonly observed among patients aged 31-40 years (37 (30.8%)). However, age distribution was not significantly associated with UAS insertion outcome (χ² = 0.856, p = 0.973) (Table 2).

**Table 2:** Association of Age With UAS Insertion Outcome (n = 120) Data are presented as number (percentage). Association between age group and UAS insertion outcome was analyzed using the Chi-squared test. Effect size was expressed as Cramer's V. Statistical significance: p-value < 0.05 was considered statistically significant. UAS: ureteral access sheath; df: degrees of freedom

Age group (years)	Failure to insert UAS (n = 16)	Successful insertion of UAS (n = 104)	Total	Chi square	df	Cramer's V	p-value
18-20	0 (0.0%)	2 (1.7%)	2 (1.7%)	0.856	5	0.085	0.973
21-30	1 (0.8%)	8 (6.7%)	9 (7.5%)				
31-40	5 (4.2%)	37 (30.8%)	42 (35.0%)				
41-50	4 (3.3%)	23 (19.2%)	27 (22.5%)				
51-60	5 (4.2%)	25 (20.8%)	30 (25.0%)				
61-70	1 (0.8%)	9 (7.5%)	10 (8.3%)				

Male patients constituted 64 (53.3%) of the study population, while female patients accounted for 56 (46.7%). Gender was also not significantly associated with UAS insertion outcome (χ² = 0.063, p = 0.802) (Table 3).

**Table 3:** Association of Gender With UAS Insertion Outcome (n = 120) Data are presented as number (percentage). Association between gender and UAS insertion outcome was analyzed using the Chi-squared test. Effect size was expressed as Cramer's V. Statistical significance: p-value < 0.05 was considered statistically significant. UAS: ureteral access sheath; df: degrees of freedom

Gender	Failure to insert UAS (n = 16)	Successful insertion of UAS (n = 104)	Total	Chi square	df	Cramer's V	p-value
Female	7 (5.8%)	49 (40.8%)	56 (46.7%)	0.063	1	0.023	0.802
Male	9 (7.5%)	55 (45.8%)	64 (53.3%)				

Comparison of anthropometric parameters revealed that patients with failed UAS insertion had significantly higher mean body weight (71.19 ± 7.08 kg vs. 58.63 ± 6.41 kg; p < 0.001) and BMI (30.38 ± 2.44 kg/m² vs. 24.66 ± 2.23 kg/m²; p < 0.001) compared with those with successful insertion. However, mean age and height did not differ significantly between the two groups (p > 0.05) (Table 4).

**Table 4:** Comparison of Anthropometric Parameters Between Study Groups (n = 120) Data are presented as mean ± standard deviation. Comparison of anthropometric parameters between study groups was analyzed using the independent samples t-test. Effect size was expressed as Cohen's d. Statistical significance: p-value < 0.05 was considered statistically significant. UAS: ureteral access sheath; BMI: body mass index; df: degrees of freedom

Parameter	Failure to insert UAS (n = 16)	Successful insertion of UAS (n = 104)	t-value	df	Cohen's d	p-value
Age (years)	46.13 ± 10.60	44.99 ± 12.11	0.34	118	0.10	0.729
Height (cm)	153.06 ± 3.71	154.15 ± 3.39	-1.18	118	0.32	0.239
Weight (kg)	71.19 ± 7.08	58.63 ± 6.41	7.20	118	1.95	<0.001
BMI (kg/m²)	30.38 ± 2.44	24.66 ± 2.23	9.42	118	2.48	<0.001

Assessment of BMI categories demonstrated that the majority of patients had normal BMI (85 (70.8%)). Failure of UAS insertion was predominantly observed among obese patients with BMI ≥ 30 kg/m² (11 (9.2%)), whereas successful insertion was most common in patients with normal BMI (84 (70.0%)). The association between BMI category and UAS insertion outcome was highly significant (χ² = 47.96, p < 0.0001) (Table 5).

**Table 5:** Association of BMI Category With UAS Insertion Outcome (n = 120) Data are presented as number (percentage). Association between BMI category and UAS insertion outcome was analyzed using the Chi-squared test. Effect size was expressed as Cramer's V. Statistical significance: p-value < 0.05 was considered statistically significant. UAS: ureteral access sheath; BMI: body mass index; df: degrees of freedom

BMI category (kg/m²)	Failure to insert UAS (n = 16)	Successful insertion of UAS (n = 104)	Total n (%)	Chi square	df	Cramer's V	p-value
18.5-24.9 (normal)	1 (0.8%)	84 (70.0%)	85 (70.8%)	47.96	2	0.632	<0.0001
25-29.9 (overweight)	4 (3.3%)	13 (10.8%)	17 (14.2%)				
≥30 (obese)	11 (9.2%)	7 (5.8%)	18 (15.0%)				

Among the study population, 69 (57.5%) patients had a tent-shaped ureteric orifice, while 51 (42.5%) had a round-shaped ureteric orifice. All failed UAS insertions occurred in patients with round-shaped ureteric orifices (16 (13.3%)), whereas no failures were observed among patients with tent-shaped ureteric orifices. This association between ureteric orifice morphology and UAS insertion outcome was highly statistically significant (χ² = 24.98, p < 0.001) (Table 6).

**Table 6:** Association of Ureteric Orifice Morphology With UAS Insertion Outcome (n = 120) Data are presented as number (percentage). Association between ureteric orifice morphology and UAS insertion outcome was analyzed using the Chi-squared test. Effect size was expressed as Cramer's V. Statistical significance: p-value < 0.05 was considered statistically significant. UAS: ureteral access sheath; df: degrees of freedom

Shape of ureteric orifice	Failure to insert UAS (n = 16)	Successful insertion of UAS (n = 104)	Total n (%)	Chi square	df	Cramer's V	p-value
Round	16 (13.3%)	35 (29.2%)	51 (42.5%)	24.98	1	0.456	<0.001
Tent-shaped	0 (0.0%)	69 (57.5%)	69 (57.5%)				

Right renal stones constituted the most common stone location (57 (47.5%)), followed by left renal stones (37 (30.8%)). Failed UAS insertion was more frequently observed in patients with right renal stones (8 (6.7%)) and right proximal ureteric stones (5 (4.2%)). The mean stone size was marginally higher in the failure group than in the success group (15.88 ± 7.76 mm vs. 13.92 ± 4.70 mm), although the difference was not statistically significant (p = 0.162). Similarly, stone density did not differ significantly between the groups (p = 0.614). Although failures appeared to occur more frequently in right-sided stones, stone location was not significantly associated with UAS insertion outcome (χ² = 4.45, p = 0.217) (Table 7).

**Table 7:** Stone Characteristics and UAS Insertion Outcome (n = 120) Continuous variables are presented as mean ± standard deviation (SD) and were compared using the independent samples t-test. Categorical variables are presented as number (percentage) and were analyzed using the Chi-squared test. Effect size was expressed as Cohen's d for stone size and stone density and as Cramer's V for stone location. Statistical significance: p-value < 0.05 was considered statistically significant. UAS: ureteral access sheath; HU: Hounsfield units; χ²: Chi-squared statistic; df: degrees of freedom

Variable		Failure to insert UAS (n = 16)	Successful insertion of UAS (n = 104)	Total	Test statistic	df	Effect size	p-value
Stone size (mm)	Mean ± SD	15.88 ± 7.76	13.92 ± 4.70	14.35 ± 5.68	t = 1.41	118	Cohen's d = 0.37	0.162
Stone density (HU)	Mean ± SD	1,377.1 ± 197.5	1,682.6 ± 2,404.3	1,478.2 ± 858.3	t = −0.51	118	Cohen's d = 0.13	0.614
Stone location	Left proximal ureter	1 (0.8%)	5 (4.2%)	6 (5.0%)	χ² = 4.45	3	Cramer's V = 0.193	0.217
	Left renal	2 (1.7%)	35 (29.2%)	37 (30.8%)				
	Right proximal ureter	5 (4.2%)	15 (12.5%)	20 (16.7%)				
	Right renal	8 (6.7%)	49 (40.8%)	57 (47.5%)				

Hydronephrosis was present in 76 (63.3%) patients and absent in 44 (36.7%). Successful UAS insertion was observed in 67 (55.8%) patients with hydronephrosis and 37 (30.8%) patients without hydronephrosis, whereas failure occurred in nine (7.5%) and seven (5.8%) patients, respectively. Hydronephrosis was not significantly associated with UAS insertion outcome (χ² = 0.399, p = 0.528) (Table 8).

**Table 8:** Association of Hydronephrosis With UAS Insertion Outcome (n = 120) Data are presented as number (percentage). Association between hydronephrosis and UAS insertion outcome was analyzed using the Chi-squared test. Effect size was expressed as Cramer's V. Statistical significance: p-value < 0.05 was considered statistically significant. UAS: ureteral access sheath; df: degrees of freedom

Hydronephrosis	Failure to insert UAS (n = 16)	Successful insertion of UAS (n = 104)	Total n (%)	Chi square	df	Cramer's V	p-value
Present	9 (7.5%)	67 (55.8%)	76 (63.3%)	0.399	1	0.058	0.528
Absent	7 (5.8%)	37 (30.8%)	44 (36.7%)				

The mean CT-measured common iliac artery diameter was significantly greater in the failure group (11.29 ± 0.91 mm) than in the success group (8.23 ± 0.63 mm; p < 0.001) (Table 9).

**Table 9:** Association of CT-Measured Common Iliac Artery Diameter With UAS Insertion Outcome (n = 120) Data are presented as mean ± standard deviation. Comparison of common iliac artery diameter between study groups was analyzed using the independent samples t-test. Effect size was expressed as Cohen's d. Statistical significance: p-value < 0.05 was considered statistically significant. UAS: ureteral access sheath; df: degrees of freedom; CT: computed tomography

Parameter	Failure to insert UAS (n = 16)	Successful insertion of UAS (n = 104)	t-value	df	Cohen's d	p-value
Common iliac artery diameter (mm)	11.29 ± 0.91	8.23 ± 0.63	16.92	118	4.46	<0.001

The mean CT-measured common iliac artery diameter increased significantly with increasing BMI, with the highest mean diameter observed in obese patients (10.33 ± 1.52 mm), followed by overweight (8.98 ± 1.67 mm) and normal BMI individuals (8.21 ± 0.58 mm) (F = 135.67, p < 0.0001) (Table 10).

**Table 10:** Association Between BMI Category and CT-Measured Common Iliac Artery Diameter Data are presented as mean ± standard deviation (SD). Association between BMI category and common iliac artery diameter was analyzed using one-way analysis of variance (ANOVA). Effect size was expressed as eta squared (η²). Statistical significance: p-value < 0.05 was considered statistically significant. BMI: body mass index; df: degrees of freedom; η²: eta squared; CT: computed tomography

BMI category (kg/m²)	Total No. of patients	Common iliac artery diameter (mm) mean ± SD	F-value	df	η²	p-value
18.5-24.9 (normal)	85	8.21 ± 0.58	135.67	2	0.699	<0.0001
25-29.9 (overweight)	17	8.98 ± 1.67				
>30 (obese)	18	10.33 ± 1.52				

## Discussion

In the present study, successful UAS insertion was achieved in 104 (86.7%) patients, while failure of insertion was observed in 16 (13.3%) patients undergoing RIRS. Comparable findings were reported by Hu et al., who documented UAS insertion failure in 29 (11.2%) patients [11]. In the present study, no statistically significant association was observed between age group and UAS insertion outcome (p = 0.973), with failures distributed across all adult age groups. Similarly, gender did not significantly influence insertion success (p = 0.802), with failure observed in seven (5.8%) women and nine (7.5%) men. Hu et al. also demonstrated no significant difference in age between successful and failed UAS insertion groups, while Damar et al. reported no meaningful association between patient sex and successful sheath placement [11,16]. These findings collectively suggest that demographic variables such as age and gender are less predictive of UAS negotiability than anatomical and anthropometric factors.

Anthropometric analysis in the present study demonstrated significantly higher body weight and BMI among patients with failed UAS insertion. The mean BMI in the failed insertion group was 30.38 ± 2.44 kg/m² compared to 24.66 ± 2.23 kg/m² in the successful group (p < 0.001). Additionally, obesity (BMI ≥ 30 kg/m²) was strongly associated with insertion failure, accounting for 11 (9.2%) failures compared with only seven (5.8%) successful insertions. Similar observations were reported by Azhar et al., who found significantly higher BMI among patients with failed UAS insertion [17]. In contrast, Hu et al. did not identify BMI as a statistically significant predictor, which may be attributable to differences in study population characteristics, ethnicity, body fat distribution, pelvic anatomy, and study methodology [11].

The present study also identified CT-measured common iliac artery diameter as a significant anatomical predictor of UAS insertion outcome, with failed insertion cases demonstrating significantly larger arterial diameter compared with successful cases (11.29 ± 0.91 mm vs. 8.23 ± 0.63 mm; p < 0.001). Hu et al. similarly reported that increased common iliac artery diameter was associated with greater difficulty during sheath advancement [11]. The common iliac artery diameter is an important radiological parameter because of its close anatomical relationship with the ureter at the pelvic brim. An increased arterial diameter may reflect greater vascular prominence and altered local pelvic anatomy, resulting in external compression or reduced mobility of the adjacent ureter. Such anatomical changes may contribute to ureteral narrowing, decreased compliance, and increased resistance during advancement of the UAS. Furthermore, common iliac artery diameter can be readily measured on routine preoperative CT scans without additional investigations, making it a practical and objective predictor of procedural difficulty. Therefore, assessment of common iliac artery diameter may help identify patients at increased risk of difficult UAS insertion and facilitate better preoperative planning, counselling, and consideration of primary ureteral stenting in selected high-risk patients. Based on the present findings, patients with obesity, round-shaped ureteric orifices, and larger CT-measured common iliac artery diameters may represent a subgroup in whom preoperative counselling and selective pre-stenting may be considered.

Among the intraoperative anatomical factors assessed, ureteric orifice morphology emerged as one of the strongest predictors of UAS insertion success. In the present study, all 16 (13.3%) failed UAS insertions occurred in patients with round-shaped ureteric orifices, whereas tent-shaped orifices were observed exclusively in successful insertion cases (69 (57.5%)) (p < 0.001). Similar findings were described by Azhar et al., who reported that a tent-shaped ureteric orifice facilitated smooth sheath advancement, while round or narrow orifices were associated with greater resistance and increased need for pre-stenting [17]. Likewise, Hu et al. emphasized distal ureteral tightness and resistance at the ureterovesical junction as major contributors to UAS insertion failure [11]. The favorable insertion outcome associated with tent-shaped ureteric orifices in the present study may be explained by improved alignment of the intramural ureter and increased ureteral compliance, permitting easier coaxial advancement of the sheath. These findings highlight the importance of careful cystoscopic evaluation of ureteric orifice morphology before sheath insertion during RIRS.

Stone-related variables did not significantly influence UAS insertion outcome in the present study. The mean stone size was slightly higher in failed insertion cases than in successful cases (15.88 ± 7.76 mm vs. 13.92 ± 4.70 mm), although the difference was not statistically significant (p = 0.162). Similarly, stone density did not differ significantly between the two groups (p = 0.614). Stone location also showed no statistically significant association with insertion outcome (p = 0.217), although failures were more frequently observed in right-sided stones. Hydronephrosis likewise demonstrated no significant association with successful UAS insertion (p = 0.528). Comparable observations were reported by Mahmood et al., Sharma et al., and Özman et al., who concluded that stone burden and stone density mainly influence operative duration and stone-free rates rather than UAS negotiability [18-20]. Although Jha et al. observed greater difficulty in UAS insertion among patients with impacted proximal ureteric calculi, the difficulty was attributed primarily to associated edema and ureteral narrowing rather than stone location itself [21]. Similarly, Hu et al. demonstrated that hydronephrosis was not a reliable predictor of sheath insertion success [11]. Collectively, these findings indicate that ureteral compliance and anatomical characteristics are more important determinants of UAS insertion success than stone-related parameters alone.

Strengths and limitations

The present study has several strengths, including its prospective observational design; performance of all procedures by a single experienced surgeon using a standardized operative protocol; comprehensive evaluation of demographic, anthropometric, stone-related, and CT-based anatomical variables; and inclusion of clinically relevant intraoperative findings such as ureteric orifice morphology and common iliac artery diameter. The study also provides valuable data from the Indian population, where literature regarding predictors of UAS insertion remains limited.

However, certain limitations should be acknowledged. The study was conducted at a single tertiary care center with a relatively modest sample size, and only 16 patients experienced failed UAS insertion, limiting the statistical power for robust multivariable predictive modelling. Patients with previous urological intervention were excluded, which may have introduced selection bias toward technically less complex cases. Although a single surgeon minimized interobserver variability, the subjective assessment of ureteric orifice morphology may limit reproducibility across institutions. Additionally, the study did not evaluate postoperative ureteral outcomes such as ureteral injury, stricture formation, or long-term functional assessment, and the findings have not been externally validated. Further large-scale multicentric studies with long-term follow-up are warranted to validate these findings, develop robust predictive models, and establish standardized risk stratification strategies for UAS insertion during RIRS.

## Conclusions

The present study demonstrates that UAS insertion during RIRS is successful in the majority of patients; however, insertion failure is significantly influenced by patient-specific anthropometric and anatomical factors. Higher body weight, obesity, round-shaped ureteric orifice morphology, and increased CT-measured common iliac artery diameter were identified as significant predictors of difficult or failed UAS placement, whereas age, gender, stone size, stone density, stone location, and hydronephrosis did not show a significant association with insertion outcome. These findings emphasize the importance of comprehensive preoperative assessment of patient anthropometry and ureteral anatomy to identify patients at increased risk of difficult UAS insertion. Such risk stratification may facilitate individualized surgical planning, appropriate patient counselling, consideration of selective pre-stenting in high-risk patients, minimization of ureteral trauma, and improved procedural safety and success during RIRS.
